# Scleral surgery for the treatment of presbyopia: where are we today?

**DOI:** 10.1186/s40662-018-0098-x

**Published:** 2018-02-26

**Authors:** AnnMarie Hipsley, Brad Hall, Karolinne M. Rocha

**Affiliations:** 1 Ace Vision Group Inc, 39655 Eureka Drive, Newark, CA 94560 USA; 2 Sengi Data, Cambridge, ON Canada; 3 Storm Eye Institute, Medical University of South Carolina, Charleston, SC USA

**Keywords:** Scleral surgery, Presbyopia, Accommodation, Presbyopia treatment

## Abstract

Presbyopia corrections traditionally have been approached with attempts to exchange power, either at the cornea or the lens planes, inducing multifocality, or altering asphericity to impact the optical system. Treatments that affect the visual axis, such as spectacle and contact lens correction, refractive surgeries, corneal onlays and inlays, and intraocular lenses are typically unable to restore true accommodation to the presbyopic eye. Their aim is instead to enhance ‘pseudoaccommodation’ by facilitating an extended depth-of-focus for which vision is sufficient. There is a true lack of technology that approaches presbyopia from a treatment based or therapy based solution, rather than a ‘vision correction’ solution that compromises other components of the optical system. Scleral surgical procedures seek to restore true accommodation combined with pseudoaccommodation and have several advantages over other more invasive options to treat presbyopia. While the theoretical justification of scleral surgical procedures remains controversial, there has nevertheless been increasing interest and evidence to support scleral surgical and therapeutic approaches to treat presbyopia. Enormous progress in scleral surgery techniques and understanding of the mechanisms of action have been achieved since the 1970s, and this remains an active area of research. In this article, we discuss the historic scleral surgical procedures, the two scleral procedures currently available, as well as an outlook of the future for the scleral surgical space for treating presbyopia.

## Background

Presbyopia means “old eye”, which is traditionally described as the gradual loss of the eye’s ability to focus on near objects due to the loss of elasticity of the crystalline lens [1–3]. Recent research, however, has demonstrated that as the eye ages there are numerous changes in other tissues of the eye such as the vitreous membrane, peripheral choroid, ciliary muscle, scleral connective tissue, and zonules, to name a few, which may contribute to the dysfunction of accommodation [4–7]. A significant consequence of aging is a progressive loss of accommodative ability, which affects an estimated half a billion people worldwide [8]. The average age of onset is 42 after which a significant and progressive decline is seen through the next two decades. A teenager has about 13 diopters (D) of subjective accommodation, whereas an average 40-year-old retains approximately 6 D and a 50-year-old 2 D [9]. According to Donder’s Curve, we lose almost 0.25 D per year throughout our 40s and 50s with an average subjective accommodation ability of 1 D by the age of 60 [9]. In wealthy nations, presbyopia correction or treatment is convenience and quality of life factor. However, in 3rd tier economic regions of the world, it is a socioeconomic burden contributing to the World Health Organization (WHO) statistics of the blindness of uncorrected refractive errors and presbyopia [10]. Lack of resources, ophthalmologists, and awareness create a culture in which the manifestation of presbyopia creates a quality life crisis with near and intermediate vision loss up to 3 D without remedy [8, 10]. In these areas, presbyopia becomes a disability and reason to leave the workforce in society. Presbyopia has an enormous impact on the gross domestic product (GDP), reducing global GDP by approximately USD 25 billion [11].

Presbyopia is typically defined following the Hemholtz theory of accommodation, wherein the loss of elasticity of the lens substance causes a reduction in accommodative ability, resulting in presbyopia [12]. As per this theory, presbyopia can be treated with spectacles, contact lenses, corneal surgery, or intraocular lenses. Spectacle and contact lens use are the conventional treatments, [13] however neither of these attempt to restore true accommodation to the presbyopic eye.

There are limitations to treating the real cause of presbyopia or the loss of accommodative ability of the lens to dynamically change focus power. Firstly, the early attempts to address presbyopia were to exchange either the power in the cornea or the lens to achieve multifocality or changes in asphericity. Corneal presbyopic correction procedures, such as presbyLASIK, attempt to create a multifocal cornea by manipulating the optical properties of the eye, asphericity, and inducing higher-order aberrations, [14] while intraocular lens (IOL) replacement may include multifocal and aspheric lenses. These vision correction procedures may compromise distance vision and degrade binocularity and stereopsis [15–17]. Performing these corrections with surgical intervention carries additional risks of regression, scarring, and night vision problems [18]. These treatments also only aim to enhance ‘pseudoaccommodation’ by facilitating an extended depth-of-focus for which vision is sufficient, [19] rather than restoring true accommodation and pseudoaccommodation together.

True accommodation is the ability of the eye to modify the focal length of the lens to see objects clearly when changing focus from distance to near. During true accommodation the ciliary muscles contract, releasing tension in the zonules, which allows the lens to return to its more natural convex shape [20]. Moreover, the ciliary muscles, the zonular tensions on the lens, and the role of the elastic choroid, in both the pre-stretch, disaccommodated state and accommodated states, all play complex roles in the amount of accommodative range and biomechanical functionality of the entire accommodation complex [5]. The biomechanics of this functional anatomy is directly proportional to the amount of accommodative amplitude and the central optical power that can be generated from the dynamic accommodative forces [4]. Moreover, as we age, there is resultant biomechanical dysfunction that is manifested with presbyopia creating a dysadaptation of binocularity, which further complicates the visual disturbances experienced with progressive presbyopia [21].

There have also been strides to classify the treatment paradigm for presbyopia, which assists the ophthalmic surgeon to determine the stages of classification of presbyopia and allow a more evidence-based decision-making tree for the treatment of presbyopia. Dysfunctional Lens Syndrome (DLS) has been described by George Waring IV and colleagues as a deterministic model to characterize the aging lens [22]. In DLS Stage I the lens becomes more rigid and less flexible, corresponding with presbyopia. In DLS Stage II contrast sensitivity loss, increased higher-order aberrations and light scatter often affect night vision function. In DLS Stage III the lens clouding is significant, and severely impacts daily activities; this stage corresponds with cataracts. Scleral surgeries are useful in as much as they can still effectuate the molding of the lens. To achieve this, the lens must be clear and void of opacities and age-related damage. The most likely candidate to achieve the most improvements with scleral surgeries would be a person who is classified as having Stage I DLS. However, candidates who are in Stage II have also received benefits from scleral procedures. Therefore, the relationship of DLS as it correlates to scleral procedure outcomes requires further investigation and remains an open question.

## Review

### Scleral surgery

#### Background

Despite the different treatment options to restore pseudoaccommodation, there remains a need for treatments to restore true accommodation combined with pseudoaccommodation to the presbyopic eye. Scleral surgical procedures have the potential to fulfill this requirement and have several advantages over other more invasive options to treat presbyopia. Firstly, scleral procedures deviate from the paradigm of ‘vision correction’ (rectifying visual acuity deficits) to a therapeutic approach; aiming to restore static and dynamic physiological function in the eye. The risk of vision loss is lower, as the cornea, visual axis, and the native crystalline lens are not involved in these procedures, which allows scleral procedures to be performed after or in combination with other corrective methods, such as cataract surgery. While their theoretical justification may be controversial [23], there has nevertheless been increasing interest in scleral surgery to treat presbyopia. In this article, we will discuss the historical scleral surgical procedures and the two procedures currently available to treat presbyopia.

#### History

Scleral surgical procedures, as a treatment for presbyopia, were followed from the surgical myopia treatments of Fyodorov in the 1970s. Fyodorov treated myopia with radial keratotomy (RK) - radial or spoke cuts through the cornea [24]. Thornton later expanded RK surgery to the sclera, using a procedure known as anterior ciliary sclerotomy (ACS) [25]. In ACS, radial incisions are not made in the cornea, but in the sclera overlaying the ciliary muscle [26]. The aim was to increase the space between the lens and the ciliary muscle, tightening the zonules and increasing accommodative ability [26]. Accommodation was observed to improve slightly with ACS. However, a myopic shift of 0.5 D was also seen [25]. The accommodative improvements were also short-term, with 0.8 D of the amplitude of accommodation remaining after 12 months [26]. To reduce this regression, Fukasaku used silicone implants in conjunction with ACS [27]. These treatment options are no longer available.

#### Scleral implants

Scleral implants are based on the accommodation model described by Schachar and colleagues [28–31]. This model describes a decreasing gap between the lens perimeter and the ciliary ring with age, due to a combination of anatomical changes, as the cause of presbyopia. This model remains controversial, as it differs from the widely accepted Hemholtz model of accommodation, [12] however it is supported by experimental evidence [7, 32].

Schachar and colleagues used scleral implants in an attempt to increase the area between the ciliary muscle and the sclera to restore accommodation. The first instances used poly[methyl methacrylate] (PMMA) rod implants to expand the sclera and were referred to as ‘scleral expansion bands’ [3, 33]. Scleral expansion bands (SEBs) did achieve some success in restoring accommodation but were ultimately retired due to mixed results and low patient satisfaction [34]. There were also events of anterior ischemia, which was not an acceptable risk for an ‘elective procedure.’ This lead to a general decrease in support and interest from the ophthalmology community and almost complete abandonment of the idea that scleral procedures were viable to treat presbyopia [23, 35].

**Fig. 1:**
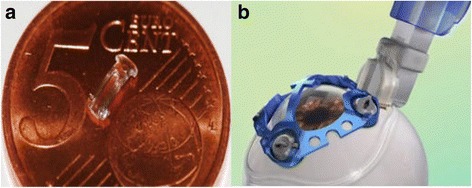
VisAbility Micro-Insert surgical procedure. **a**) VisAbility Micro-Insert; **b**) Sclerotome and docking station creating a partial thickness tunnel in the sclera. (Images courtesy of Refocus Group, Dallas, USA)

Despite early failures, using implants to expand the area between the sclera and the ciliary muscle is still an active area of research. The VisAbility Micro-Insert scleral implant (Refocus Group, Dallas, TX, USA), an updated version of the PresView (Refocus Group, Dallas, TX, USA), remains the only scleral implant with the CE mark and is currently undergoing FDA clinical trials [36]. The procedure uses four PMMA injection molded implants, each about the size of a grain of rice (Fig. 1). The implants are placed about 3000-4000 μm from the limbus and to a depth of 400 μm within the sclera. Patients are placed under monitored anesthesia care for the duration of the procedure, approximately 1 h bilaterally. The implants aim to lift the sclera and the ciliary muscle to tighten the zonular fibers holding the lens [37]. Results from a previous 24-month clinical trial with the VisAbility Micro-Insert were presented in 2013 [37, 38]. The authors subjectively evaluated the visual function of 80 patients after 24 months using a questionnaire. The participants were asked to describe their unaided vision as ‘excellent’, ‘acceptable’, or ‘poor’, pre and postoperatively. The percentage of patients reporting at least ‘acceptable’ vision after 24 months was 73% overall, and 99% for distance tasks [37, 38]. Preoperatively, 4% of patients reported at least ‘acceptable’ vision when reading newspapers, which improved to 76% of patients 24 months postoperatively [37, 38]. Approximately 83% of patients were able to complete near tasks (such as reading newspapers, prices, and medicine labels) without using reading spectacles [37, 38]. Distance-corrected near visual acuity (DCNVA) data from the same clinical trial were presented in 2014 [39]. The results showed that 93% of patient eyes had DCNVA of 0.3 logMAR (20/40 Snellen) or better [39].

While the early VisAbility clinical trial results seem promising, there are substantial risks involved for patients undergoing this procedure. Anterior segment ischemia (ASI) due to mechanical vascular compression from the implant can occur, as can subconjunctival erosion, moderate to severe subconjunctival hemorrhage, implant infection, and endophthalmitis. There is also a significant risk that the implants may become displaced [40]. An early US Federal Drug Administration (FDA) study showed that about 75% patients with the first generation of the now VisAbility Micro-Insert implant had at least one implant move or displace [40]. Other treatment options exist that may be safer for patients.

#### Scleral laser excision

Scleral laser excision procedures as a treatment for presbyopia began with Lin in 1998 [41]. Lin argued that ACS was unsuccessful due to the rapid healing of the sclera and proposed instead to ablate nearly the full thickness of the sclera [42]. Termed laser presbyopia reversal (LAPR), Lin’s surgical procedure involved radial sclerectomy with an erbium-doped yttrium aluminum garnet (Er:YAG) laser. Excisions were performed to a depth of 500-600 μm, with a length of approximately 4500 μm, and a width of 600-700 μm [42]. Results after 12 months showed 2 D of subjective accommodation. However, this may be explained by the decrease in anterior chamber depth causing a myopic shift. This treatment option is no longer available.

#### Scleral laser micro-excision

Scleral laser anterior ciliary excision (LaserACE, Ace Vision Group, Newark, CA, USA) is the only scleral laser micro-excision procedure currently available and has recently completed phase III clinical trials [43]. LaserACE is not based on the Schachar model but instead follows from VisioDynamics theory, which is a biomechanical model for the aging eye [44]. VisioDynamics theory contends that presbyopia is not a refractive error or the loss of accommodation solely, but rather an aging disease limited by structural/mechanical, extracellular and intracellular, and physiological aspects of the eye. It argues that as the eye ages, the connective tissues within begin to change and impact ocular biomechanical efficiency. This, in turn, influences visual function and ocular physiology including ocular metabolic efficiency, and ocular biotransport. A better approach to treat presbyopia may be to address these age-related changes rather than to increase scleral diameter across the globe since increasing scleral diameter could induce unwanted biomechanical effects [45].

**Fig. 2:**
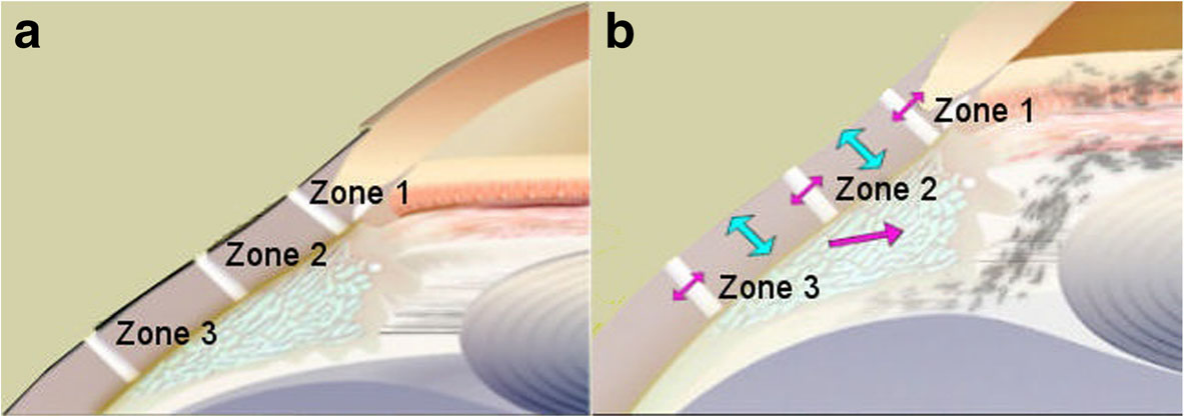
LaserACE surgical procedure. **a**) The three critical zones of significance as measured from the anatomical limbus; **b**) Restored mechanical efficiency and improved biomechanical mobility (procedure objectives). Reprinted with permission from [50]

Given the complexity of the accommodation mechanism, the Helmholtz theory is an incomplete explanation for presbyopia. Recent evidence has highlighted aging-related changes in the vitreous membrane, peripheral choroid, ciliary muscle, and zonules [4–7]. The sclera itself is known to be affected by age, bowing inward [6]. Ocular rigidity and increasing stiffness of the zonular apparatus may also further contribute to presbyopia [46, 47]. Proprioceptors in the vitreous zonular system have also been found to contribute to the loss of accommodation with age [48]. Given the many suggested contributions to the loss of accommodation, presbyopia may be better described by age-related changes in resting muscle apex thickness and accommodative lens thickening together [2]. LaserACE was thus designed to both alter the biomechanical properties of ocular tissue and improve the efficiency of the accommodation apparatus (Fig. 2).

**Fig. 3:**
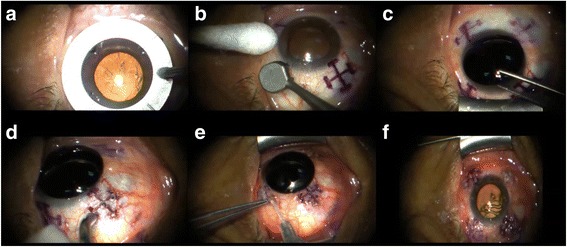
LaserACE surgical technique. Photo **a**) Quadrant marker; **b**) Matrix marker; **c**) Corneal Shield; **d**) LaserACE micropore ablation; **e**) Subconjunctival Collagen **f**) Completed 4 quadrants. Reprinted with permission from [50]

**Fig. 4:**
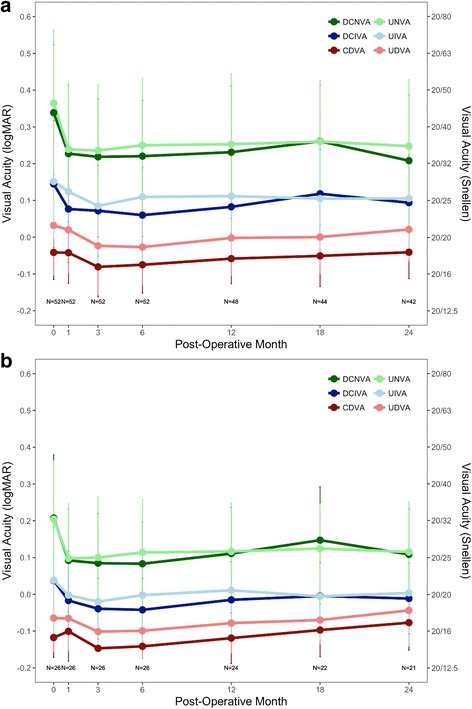
Uncorrected (lightly colored) and distance-corrected (darkly colored) visual acuity at a distance 4 m, intermediate (60 cm), and near (40 cm) for **a**) Monocular and **b**) Binocular patient eyes. Error bars represent mean ± SD. Reprinted with permission from [50]

**Fig. 5:**
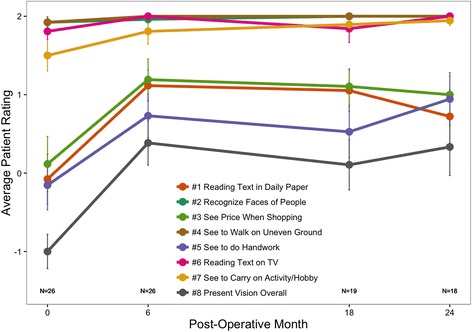
Average participant ratings from the CatQuest 9SF survey. Responses ranged from + 2, indicating no difficulty, to −2, indicating great difficulty. Error bars represent mean ± SE. Reprinted with permission from [50]

**Fig. 6:**
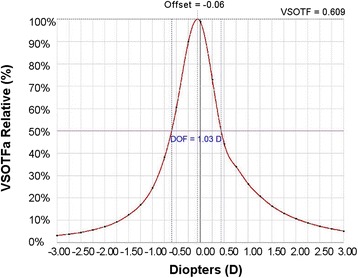
A representative figure of the depth of focus (DoF). Visual Strehl ratio based upon the optical transfer function (VSOTF) is computed as a function of defocus using a through-focus curve

**Fig. 7:**
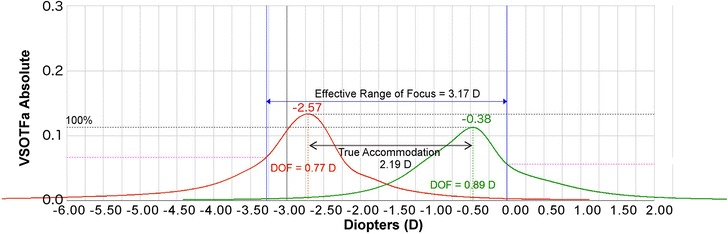
A representative figure of the effective range of focus (EROF) for a young (32-year-old) eye. Visual Strehl ratio based upon the optical transfer function (VSOTF) is computed as a function of defocus using a through-focus curve. Through-focus curves are shown for distance (green) and near (red)

**Fig. 8:**
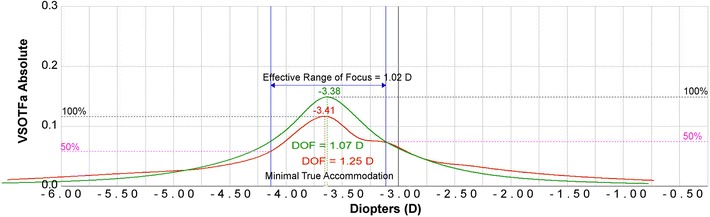
A representative figure of the effective range of focus (EROF) for an old (59-year-old) eye. Visual Strehl ratio based upon the optical transfer function (VSOTF) is computed as a function of defocus using a through-focus curve. Through-focus curves are shown for distance (green) and near (red)

**Fig. 9:**
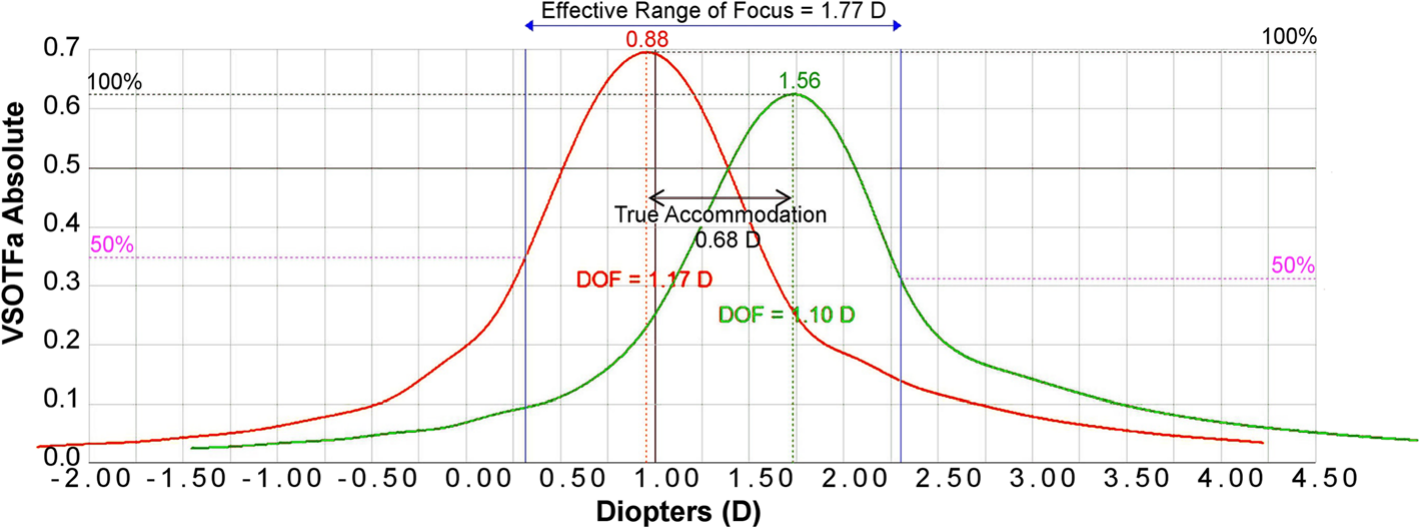
A representative figure of the effective range of focus (EROF) for a patient eye (60-year-old; 103-OD) after LaserACE. Visual Strehl ratio based upon the optical transfer function (VSOTF) is computed as a function of defocus using a through-focus curve. Through-focus curves are shown for distance (green) and near (red). Reprinted with permission from [56]

The LaserACE surgical technique is shown in Fig. 3. In brief, an Er:YAG laser is used to create a matrix array of micro-excisions (micropores, 600 μm in diameter) in the sclera, to a depth of 85-90% the thickness of the sclera (approximately 500-700 μm). The micro-excisions are done in four oblique quadrants of the eye over three critical zones of anatomical and physiological significance [44, 46, 49]. The procedure is performed under topical anesthesia and takes approximately 10 min per eye. The 3 critical zones of anatomic and physiologic importance are as follows and range from 0.5 mm up to 6.0 mm from the anatomical limbus (AL): 1) the scleral spur at the origin of the ciliary muscle (0.5 - 1.1 mm from AL); 2) the mid ciliary muscle body (1.1 – 4.9 mm from AL); and 3) insertion of the longitudinal muscle fibers of the ciliary, just anterior to the ora serrata at the insertion of the posterior vitreous zonules (4.9 – 5.5 mm from AL) [44, 46, 49]. Within the matrix, there are areas of both positive stiffness (remaining interstitial tissue) and negative stiffness (removed tissue or micropores). The differential stiffness created in these areas increases the plasticity and compliance of the scleral tissue during contraction of the ciliary muscles, and thus improve the efficiency of the accommodation apparatus.

The primary risk factor with LaserACE is accidental micro-perforation of the sclera. This can be mitigated with a collagen biomatrix. If a micro-perforation does occur, intraocular pressure may be temporarily lowered. Non-persistent mild subconjunctival hemorrhages are also a risk factor.

Data from a 24-month postoperative follow-up of the LaserACE clinical study were published in 2017 and show promising results [50]. Visual acuity at distance (4 m), intermediate (60 cm), and near (40 cm) was measured using standard Early Treatment Diabetic Retinopathy Study (ETDRS) charts, and statistical analysis was done using an ANOVA and Tukey HSD test (Fig. 4). Monocular uncorrected near visual acuity (UNVA) improved from + 0.36 ± 0.20 logMAR preoperatively, to + 0.25 ± 0.18 logMAR (*p* < 0.00005) at 24 months postoperatively, and binocular DCNVA improved from + 0.21 ± 0.17 logMAR preoperatively, to + 0.11 ± 0.12 logMAR at 24 months (*p* = 0.00026). DCNVA was also 0.2 logMAR (20/32 Snellen) or better in 83% of patients at 24 months postoperatively [50]. Stereoacuity (Randot stereoscopic test) also improved, averaging 58.8 ± 22.9 s of arc at 24 months postoperatively compared to 75.8 ± 29.3 s of arc preoperatively [50]. There were no complications such as loss of best-corrected visual acuity, cystoid macular edema, or persistent hypotony. Patients surveyed using the CatQuest 9SF Survey, [51] indicated reduced difficulty in areas of near vision, such as seeing when doing handwork, reading newsprint text, and seeing prices while shopping (Fig. 5). Patients indicated overall satisfaction with the procedure and their mean satisfaction scores significantly improved from − 1.00 (SE = 0.22) preoperatively, to + 0.33 (SE = 0.36) at 24 months postoperatively (*p* = 0.000016).

With advances in diagnostic techniques, such as wavefront aberrometry, it is now possible to objectively assess visual performance. Visual Strehl of the Optical Transfer Function (VSOTF) is an optical wavefront error-derived metric that predicts patient visual acuity [52]. It is defined as [53]:

$$ \mathrm{VSOTF}=\frac{\mathrm{the}\ \mathrm{area}\ \mathrm{under}\ \mathrm{the}\ \mathrm{contrast}\ \mathrm{sensitivity}\hbox{-} \mathrm{weighted}\ \mathrm{optical}\ \mathrm{transfer}\ \mathrm{function}}{\mathrm{the}\ \mathrm{area}\ \mathrm{under}\ \mathrm{the}\ \mathrm{contrast}\ \mathrm{sensitivity}\hbox{-} \mathrm{weighted}\ \mathrm{optical}\ \mathrm{transfer}\ \mathrm{function}\ \mathrm{for}\ \mathrm{a}\ \mathrm{diffraction}\hbox{-} \mathrm{limited}\ \mathrm{eye}} $$

A VSOTF of 0.12 correlates to approximately 0.2 logMAR, while a VSOTF of 0.3 correlates to approximately 0 logMAR [54]. VSOTF can be computed as a function of defocus by creating a through-focus curve. For example, in Fig. 6 VSOTF was determined using a ray-tracing aberrometer (Tracey Technologies, Dallas, TX, USA). A through-focus curve and VSOTF can be used to determine the objective depth of focus (DoF). A certain threshold of image quality was selected, 50% of the maximum VSOTF as used previously, [55] then the diopter range between the two points on the curve at the threshold value gives the objective DoF (Fig. 6).

Ray-tracing aberrometry objectively compares refraction and higher order aberrations at a distance and near target and can be used to determine true accommodation, effective range of focus (EROF), and pseudoaccommodation. The EROF is the range of focus with acceptable blur and includes both the true accommodation and pseudoaccommodation. Figure 7 is an example through focus curve of a young person who can still demonstrate true accommodation. Figure 7 shows near (40 cm; red) and distance (4 m; green) through-focus curves from two different wave-front scans for a 32-year-old eye. Under the through-focus curves is the ray-tracing refraction at the peak of each curve. Additionally, Fig. 7 shows the EROF, 3.17 D, which is the difference in diopters between the near and distance DoF curves at the threshold value (50% of the VSOTF). The true accommodation is equal to the difference in the spherical equivalent of ray-tracing refraction for the distance and near DoF curves (measured from curve peak to curve peak). In this 32-year-old eye, this is equal to [− 0.38 D minus (− 2.57 D)] or 2.19 D. The pseudoaccommodation is the EROF minus the true accommodation, or 0.98 D in this exam. Alternatively, Fig. 8 shows two through-focus curves from two different wavefront scans (near in red and distance in green) from a representative 59-year-old presbyopic patient. In this example, the EROF is 1.02 D, the true accommodation is minor at 0.03 D, and the pseudoaccommodation is 0.99 D, which is a typical amount of physiological accommodation that we would expect to find in a person of this age with advanced presbyopia.

In another study [56], ray-tracing aberrometry was used to produce through focus curves from two different scans to objectively evaluate the amount of combined pseudoaccommodation and accommodation in presbyopes, and their visual performance, of patients who were treated with LaserACE procedure up to 13 years postoperatively. Patient demographics and visual performance are shown in Table 1. Figure 9 shows the DoF for near (red) and distance (green) and the EROF measurements for one postoperative patient eye. The VSOTF, DoF, EROF, and objective accommodation were determined as described above and shown in Table 1. Pupil contraction can enhance DoF measurements. Figures 7, 8 and 9 show that patient pupils did contract for near observation. Since LaserACE does not affect pupil contraction, this will occur during both preoperative and postoperative accommodation measurements and can be eliminated by comparing the ranges of accommodation. The effective range of focus averaged 1.56 ± 0.36 D for all patient eyes (*n* = 6), which was higher than preoperative clinical accommodation averaging 0.92 ± 0.61 D. Patients’ DoF also increased by 0.84 ± 0.74 D compared to preoperative DoF. Up to 13 years postoperatively, true accommodation and pseudoaccommodation averaged 0.23 ± 0.24 D and 1.33 ± 0.38 D respectively. A one quarter diopter increase in true accommodation corresponds to a one-line improvement in near visual acuity. Pseudoaccommodation also improved by approximately one-quarter diopter. Up to 13 years postoperatively, the 0.5 D of restored accommodation after LaserACE was clinically significant, and there was a corresponding increase in UNVA. UNVA was 20/20 or better in 66% of patient eyes up to 13 years postoperatively. Post-operative uncorrected distance visual acuity was 20/40 or better in all patient eyes, while 83% of eyes had + 1.25 D of sphere or greater. It is possible that these patients may have latent hyperopia, and thus the restored accommodative ability after LaserACE can correct a small degree of the hyperopia in these patients improving their distance vision [57]. Previous studies have shown a similar result in hyperopic patient eyes after LaserACE [49, 50]. Patient postoperative visual acuities are shown in Table 2.

**Table 1:** Patient demographics and visual outcomes prior to and after LaserACE procedure

Patient	Age LaserACE (years)	Age Long-Term Exam (years)	Years Since LaserACE Procedure	Pre-OP MRSE	Post-OP MRSE (Long-Term)	Eye	Post-OP Sphere (D)	Post-OP Cylinder (D)	Post-OP Axis (degrees)	Post-OP IOP (mmHg)	Post-OP VSOTF	Pre-OP Depth of Focus (D)	Post-OP Depth of Focus (D)	Pre-OP Clinical Accommodation (D)	Post-OP True Accommodation (D)	Post-OP Pseudo-accommodation (D)	Post-OP Effective Range of Focus (D)
101	49	59	10	20/20	20/25-3	OD	+2.12	−1.12	150	12	0.555	0.5	1.23	0.5	0.31	1.14	1.45
						OS	+2.37	−1.12	31	14	0.281	0.5	2.82	0.5	0.06	2.10	2.16
102	48	59	13	20/20	20/20-2	OD	+1.75	−0.75	178	14	0.616	1.07	1.32	1.7	0.06	1.21	1.27
						OS	+2.00	−0.25	166	13	0.426	0.75	1.44	1.7	0.19	1.30	1.49
103	52	60	8	20/20	20/15-3	OD	+1.25	−0.50	151	11	0.609	0.5	1.03	0.5	0.68	1.09	1.77
						OS	+0.25	−0.25	14	17	0.405	0.6	1.12	0.6	0.06	1.14	1.20

*LaserACE=* laser anterior ciliary excision; *MRSE=* manifest refraction spherical equivalent; *IOP=* intraocular pressure; *VSOTF=* visual Strehl ratio based on the optical transfer function

**Table 2:** Patient long-term visual acuity after LaserACE procedure

Patient	Years After LaserACE	Eye	UDVA	UIVA	UNVA	CDVA	DCIVA	DCNVA
101	10	OD	20/25-3	20/40-2	20/60	20/20	20/20	20/20
		OS	20/40-2	20/40	20/40	20/15	20/20	20/20
102	13	OD	20/20-2	20/20-2	20/20-1	20/15	20/20	20/20
		OS	20/25	20/20-2	20/20-2	20/15	20/20	20/20
103	8	OD	20/15-3	20/20	20/20+1	20/15	20/20	20/20+1
		OS	20/20-2	20/20	20/20+1	20/15	20/20+1	20/20+1

*LaserACE=* laser anterior ciliary excision; *UDVA=* uncorrected distance visual acuity; *UIVA=* uncorrected intermediate visual acuity; *UNVA=* uncorrected near visual acuity; *CDVA=* corrected distance visual acuity; *DCIVA=* distance corrected intermediate visual acuity; *DCNVA=* distance corrected near visual acuity

DCNVA for all patients remained at 0 logMAR (20/20 Snellen) or better up to 13 years postoperatively. It is interesting to note that these patients all had prior laser vision correction (LVC) to correct their distance refraction to emmetropia before LaserACE. Since LaserACE does not touch the visual axis, these patients were able to achieve efficient near visual performance dynamically through combined accommodation and pseudoaccommodation without affecting their previous LVC.

LaserACE has many benefits compared to other presbyopia treatments. Patients experience an increased quality of life by decreasing their dependence on spectacles and contact lenses. The optical elements of the eye (cornea, lens, anterior chamber, and retina) remain untouched, unlike corneal surgical procedures. Asphericity of the eye is not manipulated, no multifocality is introduced, and the resting geometry of the eye is maintained. Furthermore, there is a physiological change in the eye improving both true accommodation and pseudoaccommodation, as well as expanding EROF. Increased dynamic movement of the lens helps facilitate ocular biotransport as well as visual function. Decreasing ocular rigidity may not only affect the development of presbyopia but also may influence the development of glaucoma and age-related macular degeneration, thus improving the longevity of the eye organ [47]. Although not available yet for North American patients, LaserACE surgery has the potential to expand EROF, restore true accommodation combined with pseudoaccommodation, and improve the quality of life in presbyopes. LaserACE is being further investigated outside of the United States.

While studies of scleral surgery as a treatment for presbyopia are ongoing, we wish to stress the importance of consistent data being collected, published, and verified using randomized multicenter studies. These studies will further clarify the role of scleral surgery and associated technologies to the treatment of presbyopia.

## Conclusions

In conclusion, scleral surgical procedures remain one of the options to restore true physiological accommodation combined with pseudoaccommodation, as well as improving effective range of focus in presbyopes. Tremendous progress in scleral surgery techniques and understanding of the mechanisms of action have been achieved since the 1970s, and this remains an active area of research. New research has identified other extralenticular factors, in addition to lens stiffness, which contributes to the loss of accommodation with age [4–7]. Utilizing this new understanding of recent research, scleral surgical procedures may be able to expand far beyond the first RK surgeries and PMMA rods used by Thornton, and Schachar and colleagues. Moreover, new diagnostic and imaging technologies allow more quantification of the results and mechanisms of these procedures such as ray-tracing, very high-frequency ultrasound biomicroscopy (VHF UBM), and high definition optical coherence tomography (HD OCT). Upon further advancement of these technologies, as well as more extensive research, a deeper understanding of this complex mechanism in the eye should be illuminated. Scleral surgeries have the potential to become the gold standard for early presbyopes who still have a clear lens due to their low invasiveness and off – optical axis appeal. This affords the potential candidates no restriction in choosing a variety of vision correction solutions for a “lifetime vision plan”, a concept that was popularized by Professor Emeritus, George O. Waring III, MD. Dr. Waring III emphasized a need for a paradigm shift from interfacing refractive patients in the scope of their vision needs through their lifetime instead of a one-time surgical candidate.
